# In search of human proteins and infectious triggers involved in periodic fever, aphthous stomatitis, pharyngitis and adenitis syndrome

**DOI:** 10.1186/1546-0096-13-S1-P21

**Published:** 2015-09-28

**Authors:** K Chisholm, A Bhatt, S Freemman, F Duke, R Fuhlbrigge, M Kenna, G Licameli, M Meyerson, S Vargas, F Dedeoglu

**Affiliations:** 1 Boston Children's Hospital, Department of Pathology, Boston, USA; 2 Stanford University, Mediciine and Genetics, Palo Alto, USA; 3 Broad Institute, Cambridge, USA; 4 Boston CHildren's Hospital, MEdicine/Division of Immunology, Boston, USA; 5 Boston Children's Hospital, Otolaryngology and Communication Disorders, Boston, USA; 6 Dana-Farber Cancer Institute, Medical Oncology, Boston, USA

## Introduction

Periodic fever, aphthous stomatitis, pharyngitis and adenitis (PFAPA) syndrome is the most prevalent pediatric autoinflammatory syndrome. For unexplained reasons tonsillectomy induces remission. The etiology of PFAPA is unknown; however, mutations of TNF receptor superfamily 1A (*TNFRSF1A*) and elevated circulating TNF-a have been described in some patients.

## Objectives

1) To identify transcriptomic or microbial signatures specific for PFAPA tonsils vs. controls; 2) to determine the presence and distribution of TNFRSF1A in tonsils of patients with PFAPA and the control population.

## Methods

Using 3 age-matched groups (6 PFAPA, 4 chronic tonsillitis and 4 obstructive sleep apnea, OSA), total RNA was extracted from tonsil punch biopsies and were subjected to massively-parallel, paired-end sequencing (~50,000,000 reads per sample) on the Illumina HiSeq platform. Resultant sequences were aligned to human and microbial reference transcriptomes in order to quantify human transcript, bacterial, and viral sequences. Tonsils from 16 children with PFAPA and 8 with streptococcus/chronic tonsillitis or OSA were stained immunohistochemically for TNFRSF1A.

## Results

Transcriptome analyses identified several genes involved in innate immune response, including TNFRSF1A, to be statistically significantly overexpressed in PFAPA tonsils. TNFRSF1A immunohistochemistry highlighted a network of dendritic cell processes extending from the basal layer of the squamous epithelium to the mantle zone of subjacent follicles. Additionally, follicles away from the squamous epithelium were lined by a similar dendritic cell network along the thin side of the mantle zone. The pattern and intensity of staining were not appreciably different between cases and controls. Computational analysis of bacterial and viral species present in PFAPA tonsils did not reveal a candidate pathogen. Unsupervised machine learning methods did not support the presence of a conserved microbial signature specific for PFAPA.

## Conclusion

Differential expression of innate immunity-related genes in PFAPA samples strengthens the hypothesis that PFAPA is mechanistically similar to other periodic fever syndromes. In this small cohort, the pattern and intensity of TNFRSF1A immunohistochemical staining were not appreciably different between cases and controls. The PFAPA tonsillar microbiome did not reveal candidate pathogens, although the study is limited by the small sample size. To our knowledge, this is the first study defining the anatomic distribution of TNFRSF1A in pediatric tonsils. TNFRSF1A is expressed in dendritic cell processes. Their localization in the interface between lymphocyte-rich squamous epithelium and subjacent germinal centers suggests that TNFRSF1A may have a role in lymphocyte trafficking to and/or from the mucosal surface.

